# The Structure of an AAV5-AAVR Complex at 2.5 Å Resolution: Implications for Cellular Entry and Immune Neutralization of AAV Gene Therapy Vectors

**DOI:** 10.3390/v12111326

**Published:** 2020-11-18

**Authors:** Mark A. Silveria, Edward E. Large, Grant M. Zane, Tommi A. White, Michael S. Chapman

**Affiliations:** 1 Department of Biochemistry, University of Missouri, Columbia, MO 65211, USA; msilveria@mail.missouri.edu (M.A.S.); largee@missouri.edu (E.E.L.); zaneg@missouri.edu (G.M.Z.); whiteto@missouri.edu (T.A.W.); 2 Electron Microscopy Core, University of Missouri, Columbia, MO 65211, USA

**Keywords:** adeno-associated virus, AAV, gene therapy, cryo-EM, electron microscopy, AAV5, receptor, PKD domain, virus structure

## Abstract

Adeno-Associated Virus is the leading vector for gene therapy. Although it is the vector for all in vivo gene therapies approved for clinical use by the US Food and Drug Administration, its biology is still not yet fully understood. It has been shown that different serotypes of AAV bind to their cellular receptor, AAVR, in different ways. Previously we have reported a 2.4Å structure of AAV2 bound to AAVR that shows ordered structure for only one of the two AAVR domains with which AAV2 interacts. In this study we present a 2.5Å resolution structure of AAV5 bound to AAVR. AAV5 binds to the first polycystic kidney disease (PKD) domain of AAVR that was not ordered in the AAV2 structure. Interactions of AAV5 with AAVR are analyzed in detail, and the implications for AAV2 binding are explored through molecular modeling. Moreover, we find that binding sites for the antibodies ADK5a, ADK5b, and 3C5 on AAV5 overlap with the binding site of AAVR. These insights provide a structural foundation for development of gene therapy agents to better evade immune neutralization without disrupting cellular entry.

## 1. Introduction

Adeno-Associated Virus (AAV) is a small (~25 nm diameter) non-enveloped single-stranded DNA virus in the *Parvoviridae* family (genus *Dependoparvovirus*) [1]. AAV was discovered in 1965 as a contaminant of simian adenovirus preparations and its replication depends on co-infection with a helper virus [2,3,4]. The icosahedral proteinaceous shell is composed of three capsid proteins. These proteins (named VP1, VP2 and VP3) share an open reading frame and differ only in their N-terminal residues. The VP1-3 capsid proteins are expressed in a respective ratio of 1:1:10 as the result of a non-canonical start site and splice variants [5]. AAV has seen success in its ability to deliver close to 5kb gene payloads to various non-dividing tissues [6] and is now arguably the leading vector for in vivo human gene therapy [7].

In recent years, recombinant AAV (rAAV) has been approved by the U.S. Food and Drug Administration (FDA) as the vector in treatments for inherited blindness (Luxterna™, 2017) and spinal muscular atrophy (Zolgensma™, 2019). These two rAAV-based drugs were developed from AAV serotypes 2 and 9 respectively [8,9]. The over ten natural AAV serotypes discovered to date have been reported to show somewhat different preferences in tissue tropism [10]. This is presumed to result from variation in capsid structure with resulting variability in binding to glycan attachment factors and protein cell entry receptors [11]. Understanding AAV-dependent tissue tropism has been a major area of research in the race to develop new rAAV gene therapy vectors.

Essential in the infection pathway of all AAVs, except the AAV4 clade [12], is the host protein AAVR [13], a cell receptor that likely plays a role in AAV’s tissue tropism [14]. Intriguingly, serotypes exhibit different binding preferences to the domains of AAVR [15,16]. The AAVR receptor is a transmembrane protein with multiple domains, among which are five tandem polycystic kidney disease-like (PKD) domains numbered in N- to C- direction as PKD1-PKD5. AAV2 binds primarily to the PKD2 domain and AAV5 to the PKD1 domain [15]. Inhibition studies with soluble single PKD domain constructs suggest that AAV2 may also bind weakly to the PKD1 domain [15], but structural studies of AAV2 with AAVR have only revealed ordered density for PKD2 [17,18], even though the receptor construct used for imaging included both domains. The clear observation of PKD2 and indiscernibility of PKD1 implies that the PKD1 domain may have multiple orientations in its complex with AAV2. When many thousands of virions are superimposed and icosahedral symmetry is applied, the signal is smeared below experimental noise levels in the averaged reconstruction. One may assume that the linker between domains is flexible, but one cannot easily distinguish a multi-Ångström continuum of PKD1 orientations from one or more discrete bound states if the equilibrium binding constant is insufficient to significantly favor one of the discrete states over others.

Here, we turn to the serotype, AAV5, which has stronger interactions with PKD1. We use a high resolution (2.5 Å) cryo-EM structure to analyze details of the molecular interactions that are presumed to be key to efficient infection and are likely to be relevant to serotype-specific tissue tropism. During our work, the EM structure of a similar complex has been published at 3.2 Å resolution [16], so the focus will be on the additional insights now possible. The nature of binding-induced conformational changes in the virus are ascertained by comparison to a structure of the unbound AAV5, determined at higher resolution (2.1 Å) than hitherto possible.

## 2. Materials and Methods

### 2.1. Expression of AAV5 and AAVR

The protein shells of AAV5, also known as virus-like particles (VLPs) were expressed in Sf9 cells using a pFastBacLIC cloning vector (addgene #30111) and the Invitrogen Bac-to-Bac expression protocol. The correct ratios of VP1, 2 and 3 were achieved, using the same mutational strategy as for the expression of AAV2 by Urabe et al. 2002 [19], with a starting sequence of ACGTCTTTTGTCGATCACCCACCCGATT (mutations highlighted in bold). Our protocol followed the analogous expression of AAV2 [18,20] except that the pFastBacLIC vector was used as a bacmid shuttle. Furthermore, with AAV5’s lower heparin affinity, AAV2’s affinity chromatography was not applicable. The AAV2 affinity chromatography purification step was replaced with three additional cesium chloride density ultracentrifugation steps for a total of six iterations instead of three. A virus-binding AAVR receptor fragment was expressed and purified as previously described [18,20]. This protein contains the two N-terminal PKD domains with an N-terminal His_6_-tag and is abbreviated PKD1-2.

### 2.2. Single Particle Cryo-EM

AAV5 and AAVR were both dialyzed into HN buffer (25 mM HEPES, 150 mM NaCl, pH = 7.4) after purification. Samples were laid on EM grids with an ultrathin continuous carbon film supported by lacey carbon on copper grids (Ted Pella, Redding, CA, USA, Cat No 01824) that were glow discharged prior to sample application. To prevent aggregation on pre-mixing AAV5 and AAVR, complex was made directly on the grid. First, 2 μL of AAV5 VLP at a VP subunit concentration of ~5.3 μM was aliquoted to the carbon side of the grid and wicked with (Whatman #1001-090) filter paper after allowing VLP to adhere to the carbon surface for 2 minutes. Then 2 μL of 33 μM PKD1-2 was added to the grid. After 30 seconds the grid was wicked again and plunge frozen into liquid ethane using an FEI Vitrobot Mark IV with a blot force of 4, time of 2 sec, temperature of 25 °C, and at 100% humidity. Native unbound VLP was prepared in a similar manner but directly plunge frozen after application.

The receptor-bound dataset was collected on a Titan Krios electron microscope (Thermo Fisher Scientific, Hillsboro, OR, USA) equipped with a Gatan K3 detector (Gatan, Inc., Pleasanton, CA, USA) and a BioQuantum Energy filter (Gatan, Inc., Pleasanton, CA, USA) operating in super resolution mode. 40-frame movies were recorded with a pixel size of 0.664Å and a total dose of 32.9 e^−^/Å^2^ with an image size of 11,520 × 8184 pixels. Defocus was randomly varied between −0.7 μm to −2.5 μm at a nominal magnification of 64,000×. A total of 734 movies were collected and were processed using RELION 3.0 (MRC Laboratory of Molecular Biology, Cambridge, UK) [21]. Movies were motion-corrected using RELION’s own implementation [21] and then contrast transfer functions (CTF) were estimated using CTFFIND4.1 (Grigorieff Lab., U. Mass, Worcester, MA, USA) [22] for each micrograph. Particle picking was first done using RELION’s LoG auto picker [21] and then picked again using RELION’s template-based auto-picking algorithm after 2D classification using the 2D class averages. Particles underwent two rounds of 2D classification (10 classes) and then an initial reconstruction was built, applying I1 symmetry. One round of 3D classification was performed (2 classes) and then the larger class, which contained over 90% of the particles, was used forthwith, and was refined into a 3D reconstruction. A mask was then applied to the model and post-processing was performed with per particle CTF estimation and per particle motion correction. Particles were then manually curated by removing particles with high astigmatism (>10%), those where the CTF could not be fit beyond 5 Å resolution, and those with estimated defocus > 3.5 μm. Finally, particles were submitted to one more round of 3D automatic refinement and masking. A local resolution map was also calculated using the RELION postprocess script [21]. Further details can be found in Table 1 and Table 2.

The unbound data was collected on a Titan Krios Electron Microscope equipped with a Gatan K3 detector operating in super resolution mode. 55-frame movies were recorded with a pixel size of 0.5295Å and a total dose of 31.7 e^−^/Å^2^ with an image size of 11,520 × 8184 pixels. Defocus was randomly varied between −0.7 μm to −2 μm at a nominal magnification of 81000×. A total of 9490 movies were collected and processed using RELION 3.1 beta [21]. Movies were motion corrected using RELION’s own implementation [21] and then CTFs were estimated using CTFFIND4.1 [22] for each micrograph. Particle picking was first done using RELION’s LoG auto picker [21] and then picked again using RELION’s template-based auto-picking algorithm after 2D classification using the 2D class averages. Particles underwent six rounds of 2D classification (20 classes) and then an initial model was built applying I1 symmetry. Particles were then automatically refined into a 3D reconstruction. A mask was then applied to the model and post-processing was performed with per particle CTF estimation and per particle motion correction. Particles were then manually curated by removing particles with astigmatism (>10%). Finally, particles were submitted to one more round of 3D automatic refinement and masking. A local resolution map was also calculated using the RELION postprocess script [21] and is illustrated in Supplementary Figure S2, along with typical micrographs in Supplementary Figure S4. Further details can be found in Table 1 and Table 2.

### 2.3. Atomic Modeling and Refinement

An atomic model of the capsid was built starting from the AAV5 crystal structure PDBid: 3NTT at 3.5Å resolution [23]. The unchanged AAV5 crystal structure was used as an external standard for calibration of the EM magnification using RSRef 0.5.6 (from the corresponding author) [24], the magnification increasing by 0.17% for the receptor-bound dataset and 0.58% for the native virus, when the reconstruction was least-squares fit to the coordinates. The starting point for the AAVR PKD1 domain was a homology model derived, using MODELLER 9.25 (Sali Lab., UCSF, San Francisco, CA, USA) [25], from nuclear magnetic resonance (NMR) structure templates PDBid: 2E7M (KIAA0319) and 2YRL (KIAA1837). The AAV5 crystal structure was docked by superimposing the icosahedral symmetry axes. Initial docking of the PKD1 domain was performed by rigid-group real-space refinement using RSRef [24] with AAV5 fixed. Manual backbone and rotamer adjustments were made using Coot 0.8.9.2 (MRC Laboratory of Molecular Biology, Cambridge, UK) [26]. Full stereo-chemically-restrained all-atom real-space refinement was performed with RSRef-embedded CNS [24,27], which was alternated with RSRef optimization of EM imaging parameters and receptor occupancy while B-factors were uniform. Before restrained refinement of atomic B-factors, the AAVR occupancy was optimized (to 0.83) using the top nine AAVR-AAV5 interacting residues for which strength of the map indicated that uniform B-factors would be a good approximation. The final model-map correlation was 0.883 for the receptor bound and 0.833 for the native virus.

Accession numbers: atomic models will be available from the protein data bank (http://www.rcsb.org) PDBid 7KP3 for AAV5 (alone) and 7KPN for the AAV5-AAVR complex. EM reconstructions will be available from the EMDataBank (http://www.ebi.ac.uk/pdbe/emdb/) with accession numbers EMD-22987 for AAV5 (alone) and EMD-22988 for the AAV5-AAVR complex.

### 2.4. Feasibility of Two-Domain Binding

Molecular modeling was used to explore the possibility that PKD1 could be bound to an AAV with the configuration found in our AAV5 structure at the same time as a PKD2 domain was bound in an AAV2-like configuration [18]. To create a hypothetical PKD1-2 two-domain model, first the PKD1 and PKD2 subunits were placed into their relative positions according to their respective structures [18]. An extended-chain linker was inserted approximately using Coot [26]. The “refine loops” tool in UCSF Chimera 1.14 (UCSF, San Francisco, CA, USA) [28] was used to run MODELLER [25] in attempts to find a stereochemically plausible connection. During model optimization, parts of the PKD1 and PKD2 domains that were clearly defined in their respective experimental EM reconstructions were anchored in position with tight harmonic restraints, such that it was only the unseen linker or disordered regions that were allowed to move. The top 5 models according to zDOPE score were evaluated and the best model was kept.

### 2.5. ELISA Binding Assays

For the virus-receptor binding assays a single PKD1 domain with an N-terminal His_6_-tag was expressed and purified in the same manner as PKD1-2. AAV5 (50 µL at 3 µg/mL in 100 mM sodium bicarbonate buffer at pH 9.6) was incubated for 1.5 h in multiple wells of a 96-well plate (Costar, Corning USA, Corning, NY, USA, cat. no. 9018). Unbound virus was then removed and the wells washed three times with Tween-20 Tris-buffered saline (TTBS; 0.05% *v*/*v* Tween-20, 50 mM Tris-HCl, 150 mM sodium chloride at pH 7.5). Following the washes, the wells were blocked with bovine serum albumin (BSA; Fisher Scientific, Hampton, NH, USA, cat. no. BP9703-100, 150 µL at 3% *w*/*v* in TTBS) and allowed to incubate for 1 h. Unbound BSA was then removed and the wells washed three times with TTBS. The ligand (see below) was added to the well and allowed to incubate for 1 h. The unbound ligand was removed and each well washed three times with TTBS. An antibody (100 µL, see below) was then added to each well and incubated for 1 h. The unbound antibody was removed and each well washed three time with TTBS. TMB ELISA substrate (90 µL, Abcam, Cambridge, UK, cat. no. ab171523) was added to each well and incubated for 5–10 minutes, during which time a yellow color developed. To stop the colorimetric reaction, 80 µL of hydrochloric acid (1 M) was added to each well and the resulting blue color was measured by reading the absorbance at 450 nm on a plate reader (Synergy H1, Biotek US, Winooski, VT, USA). All incubation steps were performed at room temperature on a rocking platform. Values were normalized by dividing by the positive control and plotted in SigmaPlot 11.0 (Systat Software, San Jose, CA, USA).

To test the competition of various concentrations of PKD1 with a constant concentration of the AAV5 antibodies ADK5a (Progen, Heidelberg, Germany, cat. no. 615148) or ADK5b (Origene, Rockville, MD, USA, cat. no. AM09121PU-N), PKD1 was diluted in buffer (25 mM HEPES, 125 mM sodium chloride at pH 7.4) containing roughly 2 nM of ADK5a or 1 nM of ADK5b to a PKD1 concentration of 6 µM. This stock was serially diluted 1:3 seven times to a final concentration of 8.2 nM PKD1 in the same buffer containing antibody. Since both of the AAV5 antibodies were derived from mice, an HRP-conjugated, goat derived, anti-mouse antibody (Alexa Fluor, Thermo Fisher Scientific, Waltham, MA, USA, cat. no. A28175) was used to determine the relative amount of AAV5 antibody present.

To test the competition of various concentrations of an AAV5 antibody (ADK5a or ADK5b) with a constant PKD1 concentration, the antibody (89 nM for ADK5a and 33 nM for ADK5b) was prepared in buffer (25 mM HEPES, 125 mM sodium chloride at pH 7.4) containing 100 nM PKD1. Seven 1:2 serial dilutions were performed using the same buffer containing 100 nM PKD1 resulting in dilutions containing antibody (0.70 nM for ADK5a and 0.26 nM for ADK5b) in the presence of 100 nM PKD1. Since PKD1 was tagged with a hexa-histidine tag, an HRP-conjugated anti-6×His antibody (Abcam, Cambridge, UK, cat. no. AB1187) was used to detect the amount of PKD1 bound in the well.

## 3. Results and Discussion

### 3.1. High-Resolution Structure of Native AAV5 and Bound AAV5-PKD12

**Figure 1:**
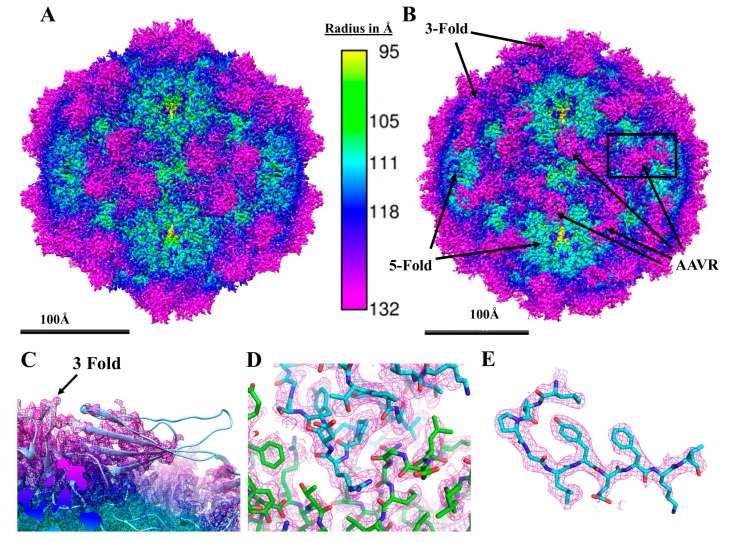
Cryo-EM reconstructions of AAV5 (**A**) and its complex with receptor AAVR (**B**–**D**). (**A**) The cryo-EM Coulombic potential map for native AAV5 at 2.1Å contoured at 1.6 sigma, colored by distance from the center of the map. (**B**) The complex of AAV5-PKD1-2 similarly rendered. The C-terminal half of the PKD1 is visible bound to each three-fold spike pointing toward the five-fold axis. (**C**) A magnified image of the outlined region in panel (**B**) with the PKD1 model built into the electron density. The Coulombic potential is strong where PKD1 is more tightly interacting with the three-fold proximal virus spike, but for the third of the domain close to the five-fold axis (right side) the map is much weaker due to presumptive disorder. (**D**) The binding site of AAV5-PKD1 shows PKD1 (blue carbon atoms) interacting with AAV5 (green carbon atoms). The coulombic potential map is shown contoured at 2.4 sigma as a violet mesh. (**E**) Detail of the PKD1 domain, showing recognizable features of the distinctive sequence _372_LTPGLYEFKV_381_ running anti-clockwise from the top.

Gold standard Fourier shell correlation (FSC) curves (Supplementary Figure S1) indicated that a 2.5 Å structure was obtained for the receptor bound dataset and a 2.1 Å structure was obtained for the native virus (Figure 1A,B). These are the highest resolutions achieved for AAV5 structures to date. When comparing both of the reconstructions, there was almost no change in the viral structure upon binding AAVR. However, in the receptor bound reconstruction, a PKD domain of AAVR was bound, at its C-terminal end, on the side of each three-fold AAV5 proximal spike. The PKD N-terminal end was disordered and pointing toward the five-fold axis of symmetry (Figure 1C). The resolution of the reconstruction is sufficient to distinguish side chains that identify the domain as PKD1 (Figure 1D,E). At the binding site, side chains and backbone carbonyl groups are clear for both AAV5 and AAVR, supporting atomic modeling with the accuracy needed to analyze the interactions between AAV5 and AAVR.

Previous studies have shown that AAV5 primarily binds to the PKD1 domain of AAVR and AAV2 binds more strongly to the PKD2 domain [15]. A two-domain AAVR construct had yielded higher resolution and clearer maps for AAVR than longer constructs in a prior cryo-EM study [18]. This is presumably due to the reduction of overall disorder with elimination of AAVR domains that do not interact directly with AAV. The increased resolution and clearer map might also result from higher AAVR binding occupancies and the absence of steric conflict between unbound receptor domains from receptor constructs bound at adjacent symmetry-equivalent sites on the viral surface. In our earlier AAV2 studies, and here with AAV5, the receptor construct contained two-domains. Both PKD1 and PKD2 domains are present in each of the EM complexes, but it is just PKD2 that is ordered enough to be visualized in the AAV2 reconstruction [18] and just PKD1 in the current AAV5 complex.

As in the prior structure [16], the PKD1 domain forms a beta-sandwich with 7 β strands that can be labeled from the amino-to-carboxyl terminus as strands A-G. It is roughly 30Å from end to end. In our reconstruction, AAVR side-chains are clearly resolved in the region close to the virus near the three-fold spike (Figure 1D,E). These side chains belong to regions of PKD1 that include the C-D loop and the E-F loop along with some residues on strand G. However, the high-resolution features become weaker and clear protein backbone lost below experimental noise around 15Å away from the binding site (Figure 1C). This makes detailed interpretation of PKD1 only possible near the three-fold and in the binding site. Modeling through the weaker regions of the map near the five-fold symmetry axis is primarily based on homology to previous structures of PKD domains, anchored by the part of the domain where the map is more ordered. The steady weakening of the map progressing towards the five-fold proximal N-terminal end of PKD1 suggests that the C-terminal end is anchored to the three-fold proximal spike of AAV5 by its interactions, but with less to restrain the N-terminal end, there might be rigid pivoting rotation about the binding site. The rigid-rotation disorder would lead to larger displacements away from the binding site at the N-terminal end of the domain without, necessarily, heightened local disorder. Indeed, the N-terminal end of PKD1, that is proximal to the virus five-fold axis, is seen when the map is lowpass-filtered to 10 Å (Supplementary Figure S3), indicating that it is not completely disordered.

The overall difference between the current structure at 2.5 Å resolution and that at 3.2 Å [16] is a surprisingly large 1.3 Å (Table 3) when coordinate frames are overlaid, as defined by the icosahedral point group symmetry. Least-squares superposition of a protomer reveals a significant rigid component that is mostly in a radial direction (0.43 of 0.67 Å translation, 0.2° rotation). This would correspond approximately to the need for a 0.4% recalibration of EM magnification, which is in the normal range of errors (see above) but went unrecognized. Following least-squares superposition, agreement of the new complex structure with the prior structure [16] is reasonable, and excellent compared with the uncomplexed AAV5 structure at 2.1 Å resolution. Improvement at high resolution is greatest in the side chains (Table 3), which is important when ascertaining virus-receptor interactions. The nine amino acids that have the most intimate interactions (Table 4) differ by a root mean square deviation (RMSD) of 1.0 Å, but with larger differences of up to 3.4 Å in some of the atoms of interacting side chains that are now seen clearly. Thus, the higher resolution structure allows potential hydrogen-bonding interactions to be examined.

Contributing to the larger differences in AAVR are several segments where the backbone differs significantly by more than 2 Å at residues 334–336 and 386–387, by up to 6 Å at 357–362, and climbing to 9.6 Å from 323 down to 312 (N-terminus of PKD2). Possible causes for some of these differences are discussed later.

PKD1 binds to AAV5 near the 3-fold symmetry axis spike, primarily to two of the variable regions (VR) in the AAV sequence that have previously been highlighted [29], namely VR-VII and a segment containing VR-IX. VR-IX, amino acids 694–713, is divergent in AAV5 with respect to most other serotypes [30]. The C–D and E–F loops in the beta sandwich that makes up PKD1 contain most of the binding interactions. These include PKD1 residues I349–G357 and L372–P374. There is one additional residue on strand G of PKD1, K399, that is also within hydrogen-bonding distance of AAV5. Potential hydrogen-bonding interactions identified by LigPlot+ 2.2 (EMBL-EBI, Hinxton, Cambridge, UK) [31] are listed in Table 4. Residues involved in hydrophobic contacts, identified by LigPlot+, are presented in Table 5.

**Figure 2:**
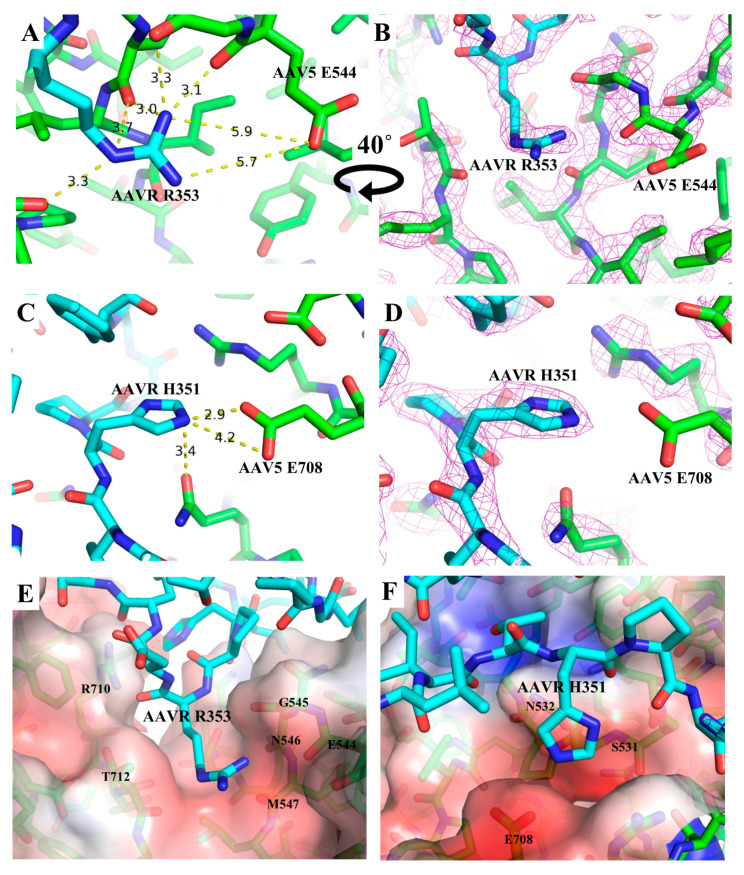
AAV5-PKD1 interactions with PKD1 carbon atoms in cyan and AAV5 carbon atoms in green. The EM map is shown at the 2 σ-level as violet mesh on panels B and D. (**A**,**B**) PKD1 R353 is shown with distances (Å) to neighboring atoms of AAV5. (**C**,**D**) PKD1 H351 is shown, highlighting potential hydrogen bonding interactions with AAV5. (**E**,**F**) The solvent accessible surface of AAV5 is colored according its electric potential as calculated using the APBS adaptive Poisson-Boltzmann solver [33]. Electro-negative surfaces (red), formed by charged side chains and polar backbone carbonyls, form pockets on AAV5 into which AAV5 R353 (E) and H351 (F) are accommodated.

The two AAVR residues, R353 and H351, are of particular importance due to their potential for polar interactions with AAV5. AAVR R353, extends from the end of PKD1 into a polar pocket between the AAV5 VR-VII and VR-IX loops. On three sides, R353 is wrapped by AAV5 backbone carbonyls within hydrogen-bonding range (Figure 2A,B; Table 4). The positively charged R353 guanidinium is extended towards a negatively charged region of the viral surface near AAV5 E544 (Figure 2E). As is common in EM maps, the carboxylate of E544 is disordered, perhaps the result of radiation damage [32], and might not be salt bridged. However, enough of the side chain is seen to know that E544 contributes to the negatively charged surface of AAV5 into which AAVR R353 fits (Figure 2E). Similarly, AAVR H351 interacts with a charged region of the AAV5 surface (Figure 2C,D,F, Table 4). The carboxylate of AAV5 E708 is similarly disordered, but it has to be in proximity with, and perhaps salt-bridged with N_ε_ of His_351_H^+^. The protonation state is implied by a second (N_δ_) to carbonyl hydrogen bond. Indeed, the histidine imidazole sits in another pocket on the AAV5 surface that is strongly negatively charged (Figure 2F).

While the interactions between AAVR and AAV5-VR-IX involve predominantly hydrogen-bonding and charge-charge interactions, the interactions between AAVR and VR-VII are less dominated by electrostatics and hydrogen-bonding. Instead they involve hydrophobic contacts formed by AAVR L376 and AAV5 Y542, L543 and A540.

At the other end of PKD1, when looking for interactions near the five-fold symmetry axis, it is only after application of a 10 Å low-pass filter that our reconstruction shows the N-terminal segment of PKD1 (Supplementary Figure S3). Attempts were made at local reconstruction [34] in the hope that the disorder resulted from discretely different configurations that could be classified. A receptor-virus symmetry mismatch could be one of several possible causes, although the disorder is near the N-terminus, remote from the PKD2 domain which could conceivably be forming symmetry-breaking interactions. Attempted local classification did not yield an improvement over the symmetrized reconstruction. One possible explanation would be that the disorder is over a continuous spectrum of local configurations, perhaps because the interactions with AAV5 near the five-fold are not strong enough to restrain AAVR to discrete orientations.

An atomic model has been proposed previously in which AAVR S319 interacts with AAV5 V305, near the five-fold symmetry axis, but it appears that this part of the model was not resolved experimentally [16]. Our 10 Å lowpass-filtered map reveals the five-fold end of PKD1 but not at sufficient resolution to trace backbone. The envelope, however, is more consistent with a path homologous to a canonical PKD domain, without the deviation required to bring AAVR into proximity with AAV5 V305 (Supplementary Figure S3). Current experimental evidence does not support the existence of strong interactions between AAVR and the region of AAV5 near the five-fold axis. The absence of such interactions is consistent with variation in receptor orientation and the observed progressively greater disorder near the N-terminal end of PKD1. Residues outside AAV5’s five-fold pore should not be expected to be conserved on account of AAVR interactions.

### 3.2. Antibody Neutralization of AAV5 and AAVR

To date, structural analyses have been performed for four complexes of antibodies with AAV5. These include two neutralizing antibodies ADK5b [35] and H2476 [36], and two non-neutralizing “binding” antibodies, ADK5a [35] and 3C5 [37]. Structures are not available in publicly accessible databases, but the potential for interference between antibody and receptor binding can be assessed approximately through overlap of footprints. This can be visualized by highlighting contact residues listed in the respective publications [35,36,37] on a Roadmap projection of the AAV5 surface [38] as calculated by RIVEM 4.5 (Xiao Lab., UT El Paso, TX, USA) [39].

**Figure 3:**
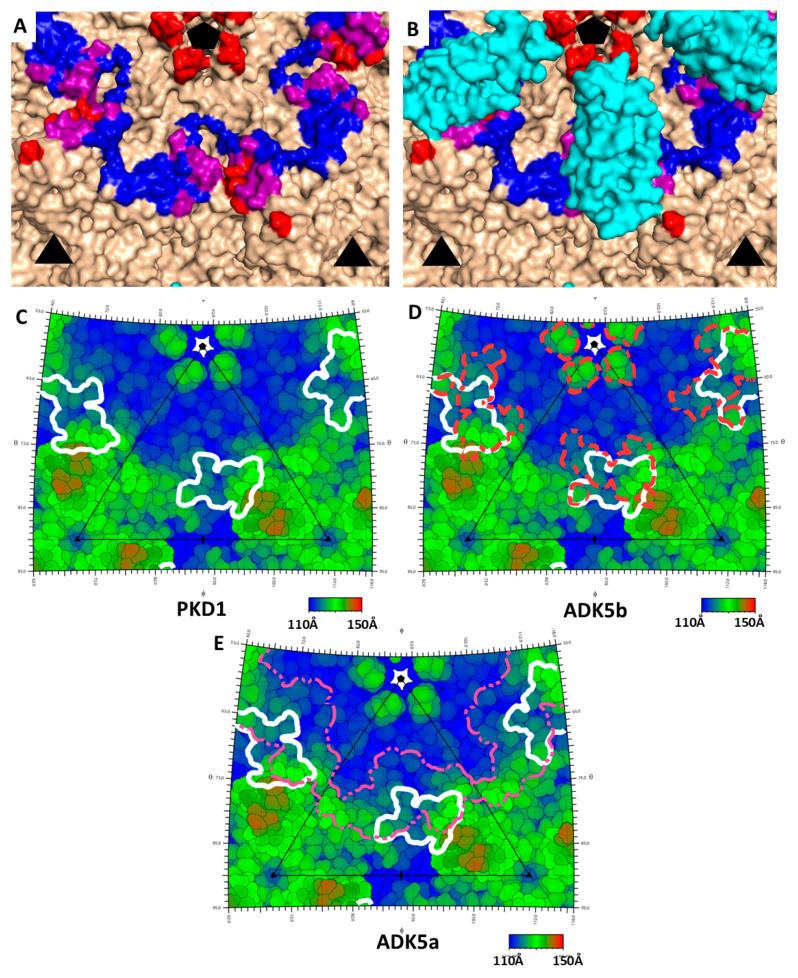
Overlap in the binding sites on AAV5 of receptor AAV5 and antibodies ADK5a & ADK5b. (**A**) The solvent accessible surface of AAV5 is colored with the ADK5a footprint blue, AKD5b red, and amino acids common to both, purple. The footprints comprise residues identified in prior EM structures at 11 Å resolution [35]. Three-fold symmetry axes, marked as triangles, map to equatorial three-folds left and right of center in Figure 1B, while the five-fold (pentagon) maps to a five-fold above center on the mid-line of Figure 1B. The view in in Figure 1B and here is down a two-fold axis. (**B**) The current structure of AAVR’s PKD1 is overlaid (with its symmetry equivalents), showing that the antibody footprints are substantially occluded. (**C**–**E**) Roadmap projections of the AAV5 surface colored by distance from the center of the virus. The perspective is the same as in panels A & B. The triangle shows one unique asymmetric unit of the surface, which, if expanded 60-fold by the icosahedral symmetry, would generate the entire virus surface. (**C**) The contact footprint of AAVR PKD1 is outlined in white. It is concentrated on the side of a three-fold spike, and although the domain occludes access to a swath leading up towards the five-fold, there is not the previously reported [16] contact near the five-fold pore. A magnified version is provided in the supplement (Figure S5) with residues labeled. (**D,E**) illustrate the footprints of ADK5b and ADK5a superimposed on that of AAVR PKD1, showing substantial overlap.

The neutralizing mechanism of ADK5b was unknown at the time that its complex with AAV5 was visualized by cryo-EM [35]. ADK5b is unique, among the four antibody complexes characterized in that its epitope is strikingly congruent with the binding footprint of AAVR PKD1 that we now visualize by cryo-EM. Indeed, the overlap is almost complete, suggestive that the molecular mechanism of ADK5b neutralization of AAV5 is likely to occur via inhibition of AAVR receptor binding (Figure 3).

**Figure 4:**
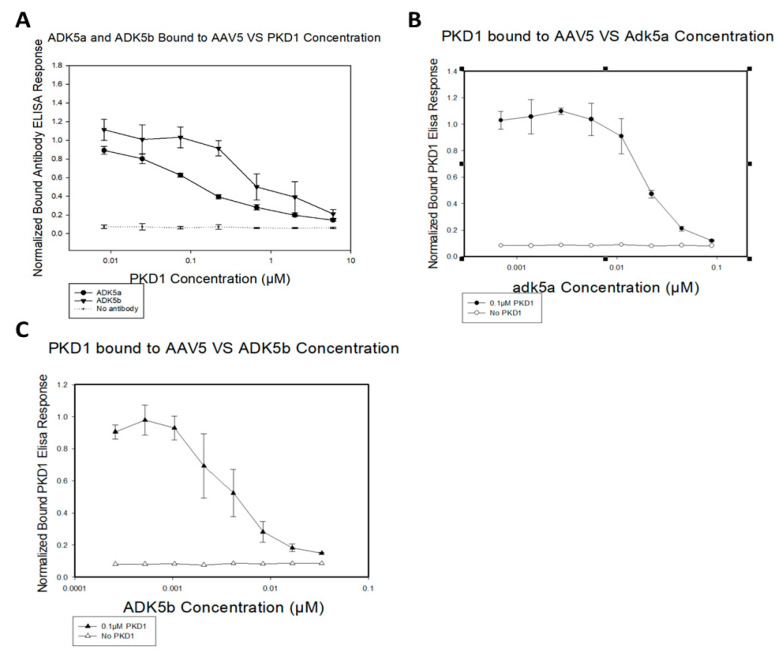
Competition assays of ADK5a, ADK5b, and PKD1. (**A**) ELISA of antibody binding to AAV5 with constant antibody concentration and differing levels of PKD1 concentration, performed as six replicates and normalized to a positive control without PKD1. (**B**) ELISA of PKD1 binding to AAV5 with a constant PKD1 concentration of 0.1µM and various levels of ADK5a performed in triplicate and normalized to a positive control without antibody. (**C**) The same as B but with ADK5b instead of ADK5a. All error bars are ± 1 standard deviation.

The binding antibody ADK5a had been classified as non-neutralizing, due to a failure to inhibit 50% of cellular transduction at levels tested [35]. Its epitope is close to that of ADK5b and the ADK5a footprint partially overlaps that of PKD1 (Figure 3B,E). The antibody sits between two PKD1 domains and its footprint suggests that it would interfere with binding to AAVR at both of the neighboring symmetry-equivalent binding sites of PKD1 (Figure 3). To verify that this overlap of ADK5a would interfere with AAVR-binding, we performed AAV5-binding competition assays of ADK5a or ADK5b with expressed AAVR PKD1 domains (Figure 4). The assays show clear competition between both antibodies and PKD1. It is noted that these experiments are performed with a minimal receptor constructed of just PKD1, and that steric conflict (and competitive inhibition) could be increased by domains (PKD2, PKD3, …) more distal from the viral surface. It is emphasized that there is not a qualitative difference between the structural analyses that could explain why ADK5b is neutralizing, while ADK5a appears not. In both cases, structures indicate extensive binding site overlap, where bound antibody would preclude binding of AAVR receptor at proximal sites, and this has been confirmed empirically through competition ELISA assay. The prior designation of ADK5a as non-neutralizing was based upon weak transduction inhibition relative to ADK5b [35]. Our competition ELISA assays indicate an ADK5a IC_50_ that is five-fold higher than ADK5b (Figure 4B,C), so the designation of neutralizing, or not, likely depends upon antibody concentration. It is noted that there are 60 symmetry-equivalent AAVR binding sites on each virus, and the level of inhibition will depend upon the degree of antibody saturation achieved, which will be concentration-dependent. It is not known whether a five-fold difference in IC_50_ is critical under physiological conditions, or is quantitatively large enough to change neutralization designation, but the results here suggest that ADK5a and ADK5b are fundamentally similar, and might both ultimately be considered to be neutralizing.

**Figure 5:**
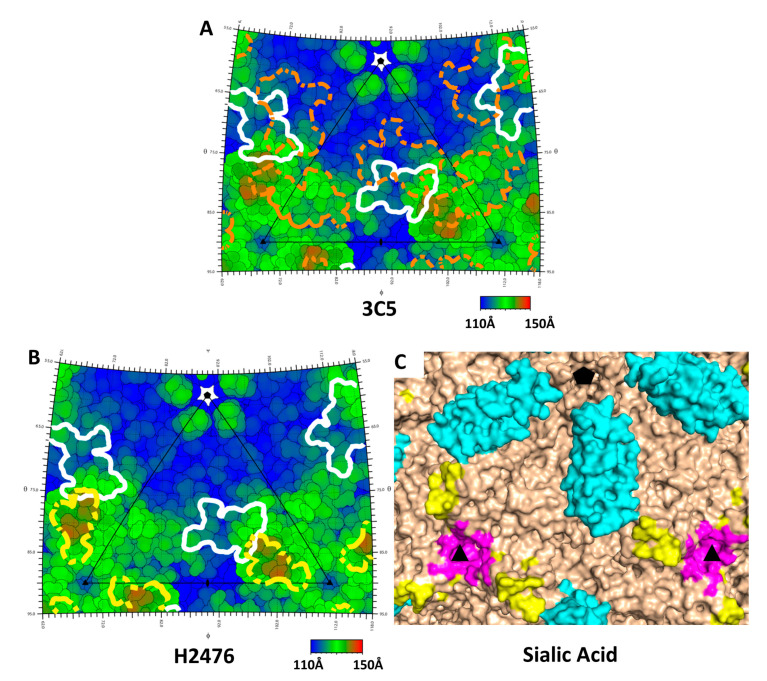
Roadmaps show footprints of PKD1 (white) with 3C5 (**A**) and H2476 (**B**) on AAV5 rendered with the same perspective as Figure 3. (**C**) A 3D surface representation of AAV5 shows the H2476 footprint (yellow) in approximately the same proximity to residues implicated in sialic acid binding attachment (magenta) and the PKD1 domain of AAVR (cyan) implicated in cell entry.

As with ADK5a, the antibody 3C5 Fab footprint partially overlaps with that of PKD1 [37] yet it does not show neutralizing ability in the FAb form. 3C5 only showed IgG neutralization at high concentrations (100,000 fold excess AB:AAV) [40]. Structural studies have found that it primarily binds to VR-I, III, V and some residues on VR-VII and that the FAb lays down tangentially on the virus surface [37]. When its footprint is displayed on a roadmap, it is clear that the tangential orientation brings part of the constant region into contact with PKD1 binding residues, and its presence would occlude the PKD1 binding site (Figure 5). The tangential orientation of the antibody may result in steric conflict of the antibody with copies of itself bound at symmetry-equivalent sites. Thus, the configuration seen might be incompatible with full antibody-virus saturation. One can speculate that the lack of transduction inhibition with an antibody that occludes receptor-binding could result from receptor sites that are left unblocked, or that antibody saturation of the virus requires a less favorable 3C5 configuration with which AAVR can compete dynamically for binding.

The hybridoma-derived neutralizing antibody H2476 had been subject to negative selection for affinity to nanobody AVB, thereby increasing the likelihood of recognizing an epitope near the three-fold symmetry axis [36]. Its footprint overlaps only slightly with that of PKD1 (Figure 5B,C), but, if coordinates for H2476 were available, greater steric conflict between the bound proteins might be apparent further away from the virus surface. H2476 also comes close to the binding site of the sialic acid (SIA), a marker of where glycosaminoglycan attachment factors are bound [41] (Figure 5C). Indeed, Jose et al. [36] proposed that H2476 may inhibit AAV5 by blocking the binding of sialic acid. Just as with AAVR, potential interference is not limited to the SIA contact residues but could involve hindrance within any of the larger glycan attachment factors terminated by the SIA. It is equally plausible that bound H2476 could be interfering with either glycan attachment or AAVR-mediated entry, and that there are at least two possible neutralization mechanisms. Indeed, it should be noted that while we are gaining a new appreciation of the number of neutralizing antibodies that could interfere with AAVR-binding in different AAV serotypes, there are other antibodies that are neutralizing in AAVR-independent ways.

### 3.3. PKD1 Binding Site on AAV2

**Figure 6:**
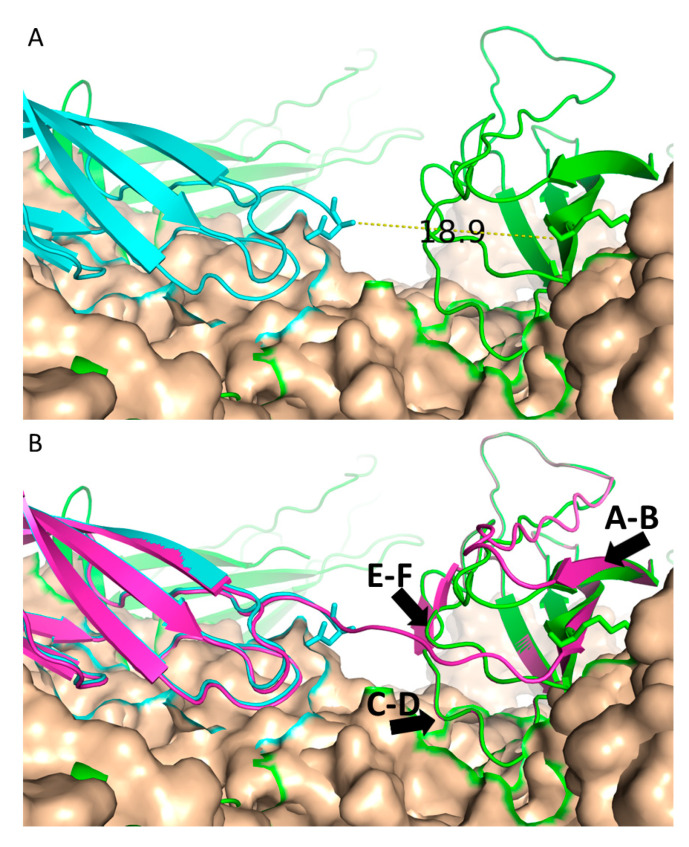
AAV5 (wheat) with PKD1 in green and PKD2 from PDB 6NZ0 overlaid in cyan. (**A**) The gap between K399 and N405 measures 19 Å. (**B**) PKD1 and PKD2 are connected in magenta with relevant loops labeled. The model in panel B has been energy minimized using MODELLER [25].

Although the contribution of PKD1 to the AAVR binding affinity of AAV2 is much less than for AAV5, evolutionary arguments might lead to an expectation that PKD1 would be binding to corresponding sites on AAV2 and AAV5. Experimental evidence has not yet emerged in support of this supposition. If the two viruses are superimposed, can the bound configurations of PKD1 and PKD2 be connected plausibly into a single polypeptide? Our cryo-EM complexes of both AAV2 and AAV5 contained a receptor construct with both PKD1 and PKD2 domains, even though only one domain is ordered enough to be resolved in each structure. In the AAV5 complex, AAVR backbone fades away beyond K399 at the C-terminal end of PKD1, and the backbone is visible in the AAV2 complex only after the inter-domain linker at N405 [18]. The gap from one domain to the other is 19 Å (Figure 6A), which could, in principle, be bridged by the five unseen linker residues if in an extended conformation. However, a direct link between the two bound domains appears implausible because it requires a stereo-chemically strained turn at K399 and displacement of the PKD1 E-F and A-B loops into conformations that have not been seen in homologs (Figure 6B). Strain persists even after relaxation by energy minimization using MODELLER [25]. There remain multiple close contacts (< 3 Å) between the inter-domain linker and the E-F loop.

While a direct connection may not be likely, another possibility is suggested by two homologous PKD domain structures in the unliganded mouse SorCS2 ectodomain (SorCS2). SorCS2 homodimers bind to nerve growth factor (NGF) to form the SorCS2-NGF complex. SorCS2 proteins form stable homodimers in solution [42,43] and the crystal structures of both the unliganded SorCS2 ectodomain and the SorCS2-NGF complex have recently been solved [43]. The unliganded and bound structures suggest the two PKD chains in SorCS2 twist around each other at the domain linkers to form a flexible head-to-tail homodimer [43]. Like SorCS2, our PKD1–2 construct is predominantly a dimer in free solution [18,20]. Although only single PKD domains have been seen in cryo-EM reconstructions, it is plausible that two-domain binding could be achieved by AAV2 (and its evolutionary ancestors) if the PKD1 and PKD2 interactions are with different chains of an oligomeric AAVR receptor. This proposed configuration would alleviate the constraint imposed by bound domains that need to be connected by a 19Å linker (Figure 6).

**Figure 7:**
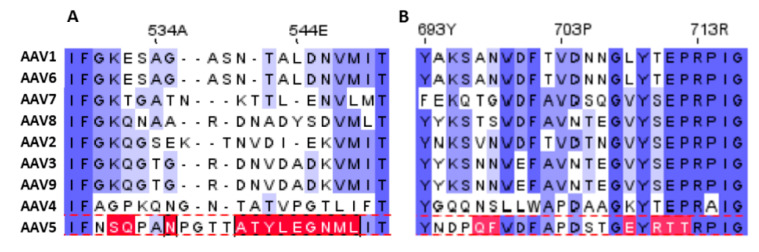
Alignment of variable regions VII (**A**) and IX (**B**) of various AAV serotypes with AAV5, done by first using Clustal Omega [44], then manually adjusted for consistency with structural superimposition. Residues that bind PKD1 are highlighted in red. This alignment shows little conservation of PKD1 binding residues from AAV5 with any other common serotype.

From an amino acid perspective there is little conservation of PKD1 binding residues seen in AAV5 with AAV2. A sequence analysis of the AAV5 binding site is shown in Figure 7. Two of 19 potentially PKD1-interacting residues are conserved in AAV2. The most noteworthy amino acid difference in VR-VII is the presence of S531 at a position that is a lysine in other serotypes (1–3 & 6–9) that are AAVR-dependent (K544 in AAV2). The lysine extends into the space that the PKD1 R353 would occupy if bound as in the AAV5 complex, potentially causing both steric and electrostatic conflicts. If the PKD1 binding site is conserved on AAV2, this conflict would need to be resolved by a conformational change of AAV2 upon binding of PKD1.

Summarizing information from several sources as it impacts our understanding of AAV2 binding to AAVR: (a) competition assays indicate that PKD1 interacts with AAV2, more weakly than PKD2, but sufficient for measurable transduction inhibition [15]; (b) not only is the PKD1 not seen in the EM structure of the AAV2 complex [18]; but binding of PKD1 in an AAV5-like manner seems (c) stereo-chemically implausible, unless PKD1 and PKD2 are from separate chains in a multimeric receptor; and (d) would require local adjustments to allow AAV5-like binding to AAV2 without side chain conflicts. It would be extraordinary if AAV serotypes had evolved convergently to bind at different sites to different domains of the same receptor. It leads us to speculate that there might have been an AAV evolutionary progenitor that did bind simultaneously to both domains in a multimer, or that both domains are bound simultaneously (only) upon an uncharacterized conformational transition in AAV, or that a dynamic change between binding states is needed during entry.

## 4. Conclusions

The high-resolution structure of the AAV5-AAVR complex is a key foundation in understanding viral entry and in gene therapy vector development. With high resolution structures of both complex and native virus, interactions can be analyzed at the atomic level. A precise understanding of the AAVR binding footprint on AAV5 has allowed comparison to previously characterized neutralizing epitopes. Such a comparison brings new understanding that, for many of the neutralizing monoclonal antibodies of both AAV2 and AAV5, there is substantial overlap between bound receptor and antibody. This has led to new experimental measurements of binding competition, and to a new appreciation that receptor-binding inhibition is, after all, a mechanism of neutralization that can be used to rationalize a number of antibodies. The structures provide a foundation for vector engineering in attempts to disrupt immune recognition while preserving core receptor interactions needed for cell entry.

## Figures and Tables

**Table 1:** Cryo-EM data collection and processing.

Data Collection	AAV5 (Uncomplexed)	AAVR-AAV5 Complex
Magnification	81,000×	64,000×
Voltage	300 kV	300 kV
Electron exposure	31.7 e^−^/Å^2^	32.9e^−^/Å^2^
Defocus range	−0.7 to −2 μm	−0.7 to −2.5 μm
Pixel size	0.530 Å	0.664 Å
(on refinement vs. atomic model:)	0.533 Å	0.665 Å
Data processing		
Motion correction	Relion 3.1-beta	Relion 3.0
CTF estimation	CTFFIND-4.1	CTFFIND-4.1
Symmetry imposed	I1	I1
Initial particle images	672,106	168,275
Final particle images	373,426	159,673
Map resolution	2.13 Å	2.5 Å
FSC threshold	0.143	0.143

**Table 2:** Refinement of the atomic model.

	AAV5 (Uncomplexed)	AAVR-AAV5 Complex
Protein atoms/asymmetric unit (mean B-factor):	4096 (18.6 Å^2^)	4794 (28.83 Å^2^)
RMS bond length deviation from ideal	0.011 Å	0.016 Å
RMS bond angle deviation from ideal	1.4°	1.8°
Ramachandran outliers	2 (0.4%)	4 (1%)
Cross-correlation (model-map)	0.833	0.883
Resolution from model-map refinement (d_0.5_)	1.91 Å	2.51 Å

**Table 3:** Comparison of structures.

Atoms from AAVR-AAV5 Complex at 2.5 Å Resolution (This Paper)	Alignment	RMSD vs. AAVR-AAV5 Complex at 3.2 Å Resolution [16]	RMSD vs. AAV5 at 2.1 Å Resolution (Uncomplexed, This Paper)
All protein atoms	By point-group symmetry	1.3 Å	0.44 Å
All protein atoms	All-atom least-squares	0.94 Å	0.43 Å
AAV5, all atoms	All-atom least-squares	0.8 Å	0.4 Å
AAV5, backbone	All-atom least-squares	0.5 Å	0.3 Å
AAV5, side chains	All-atom least-squares	1.0 Å	0.5 Å
AAVR (PKD1), all atoms	All-atom least-squares	2.3 Å	n/a
AAVR (PKD1), backbone	All-atom least-squares	1.9 Å	n/a
AAVR (PKD1), side chains	All-atom least-squares	2.6 Å	n/a

**Table 4:** Potential AAVR-AAV5 intermolecular hydrogen bonds and salt bridges.

AAVR	AAV5	Donor to Acceptor Distance (Å)
Ile_349_CO	Gln_532_N_ε_	3.1
His_351_N_ε_	Glu_532_O_ε_	3.4
Arg_353_N_ε_	Thr_712_CO	3.3
Arg_353_N_η_	Gly_545_CO	3.3
Arg_353_N_η_	Glu_544_CO	3.1
Arg_353_NH	Gly_545_CO	3.2
Arg_353_NH	Met_547_CO	3.0
Arg_353_CO	Arg_710_N_ε_	3.2
Tyr_355_CO	Arg_710_N_ε_	2.4
Lys_371_N_ζ_	Gln_697_O_ε_	2.3
Leu_376_NH	Asn_546_O_δ_	2.9
Leu_376_CO	Asn_546_N_δ_	3.1

**Table 5:** Hydrophobic contacts between AAVR and AAV5.

	**AAVR Residues Involved in Hydrophobic Contacts with AAV5**									
	Thr_350_	Pro_352_	Asp_354_	Ser_356_	Pro_374_	Gly_375_	Leu_376_	Thr_397_	Lys_399_	
	**AAV5 Residues Involved in Hydrophobic Contacts with AAVR**									
Asn_442_	Ser_531_	Ala_540_	Thr_541_	Tyr_542_	Leu_543_	Asn_546_	Met_547_	Leu_548_	Gln_697_	Phe_698_

## Supplementary Materials

The following are available online at https://www.mdpi.com/1999-4915/12/11/1326/s1, Figure S1: Fourier Shell correlation (FSC) curves from the Relion program Postprocess, Figure S2: Coulombic potential maps representing local resolution, Figure S3: The 10 Å lowpass filtered cryo-EM coulombic potential map for AAV5 in complex with PKD1-2, Figure S4: Typical Micrographs from the datasets, Figure S5: A full sized roadmap of PKD1 footprints on AAV5.
