# Supplementary material for: The Structure of an AAV5-AAVR Complex at 2.5 Å Resolution: Implications for Cellular Entry and Immune Neutralization of AAV Gene Therapy Vectors
Source: Viruses. 2020 Nov 18;12(11):1326. doi: 10.3390/v12111326 (PMC7698955; doi:10.3390/v12111326)
Supplement: Supplementary file 1 [file viruses-12-01326-s001.pdf]

viruses

Article

The Structure of an AAV5-AAVR Complex at 2.5 Å Resolution: Implications for Cellular and Immune Neutralization of AAV Gene Therapy Vectors Supplementary Materials

Mark A. Silveria[1], Edward E. Large[1], Grant M. Zane[1], Tommi A. White[1,2] and Michael S. Chapman[1]*

[1] Department of Biochemistry, University of Missouri, Columbia, MO 65211, USA; msilveria@mail.missouri.edu (M.A.S.); largee@missouri.edu (E.E.L.); zaneg@missouri.edu (G.Z.); whiteto@missouri.edu (T.W.); chapmanms@missouri.edu (M.S.C.)
[2] Electron Microscopy Core, University of Missouri, Columbia, MO 65211, USA
* Correspondence: chapmanms@missouri.edu; Tel.: +1-573-882-9825

Received: 04 September 2020; Accepted: 14 November 2020; Published: date

Supplementary Figure S1

A

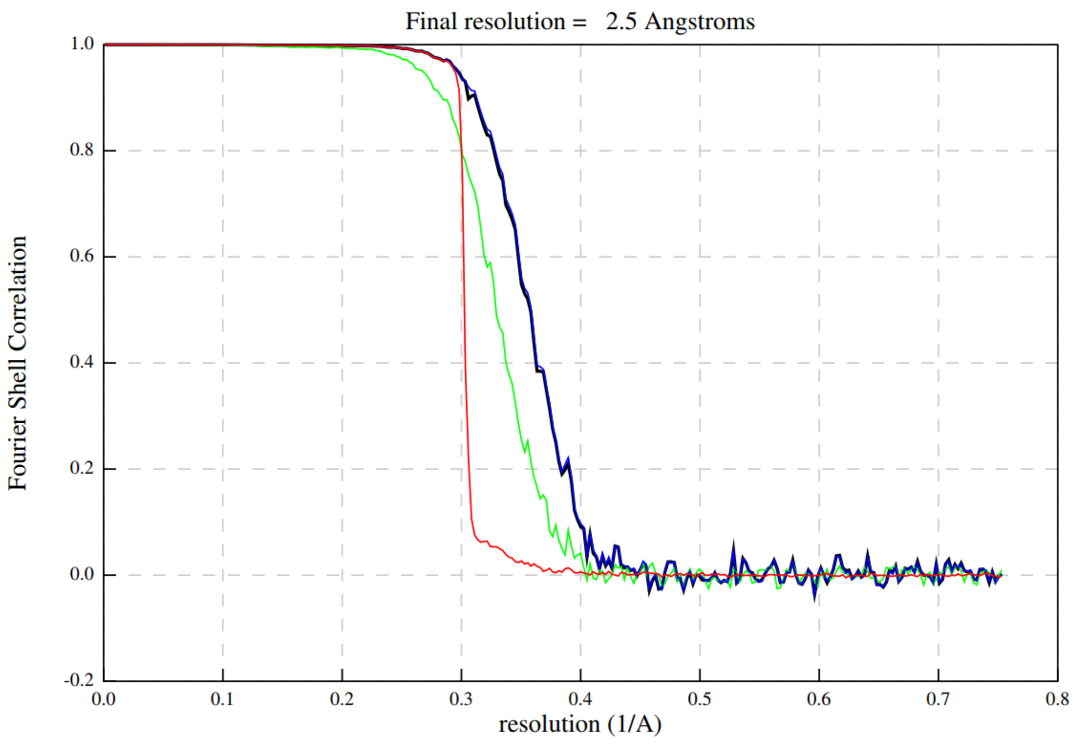

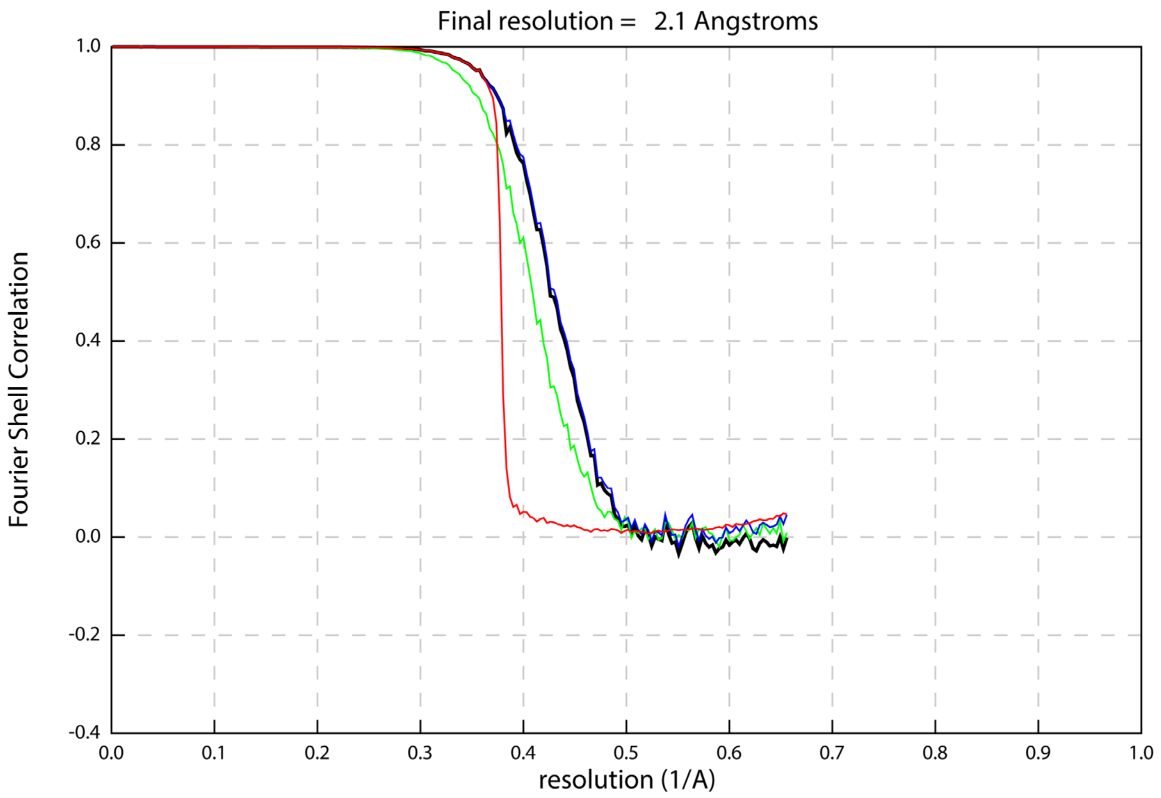

Figure S1. Fourier Shell correlation (FSC) curves from the Relion program Postprocess [1] for AAV5 reconstructions: (**A**) The AAVR-bound dataset; and (**B**) uncomplexed AAV5, showing the final resolution of the EM maps. FSC curves are shown for the unmasked map (green), the masked map (blue), the maps with Fourier components ≤ 3.32 and 3.08 Å replaced by random noise and then masked for A and B respectively (red), the masked corrected map (black) which shows the true resolution by factoring potential resolution artifacts due to masking. This is done by subtracting the random noise FSC from the masked FSC and dividing by 1 minus the random noise FSC [2].

Supplementary Figure S2

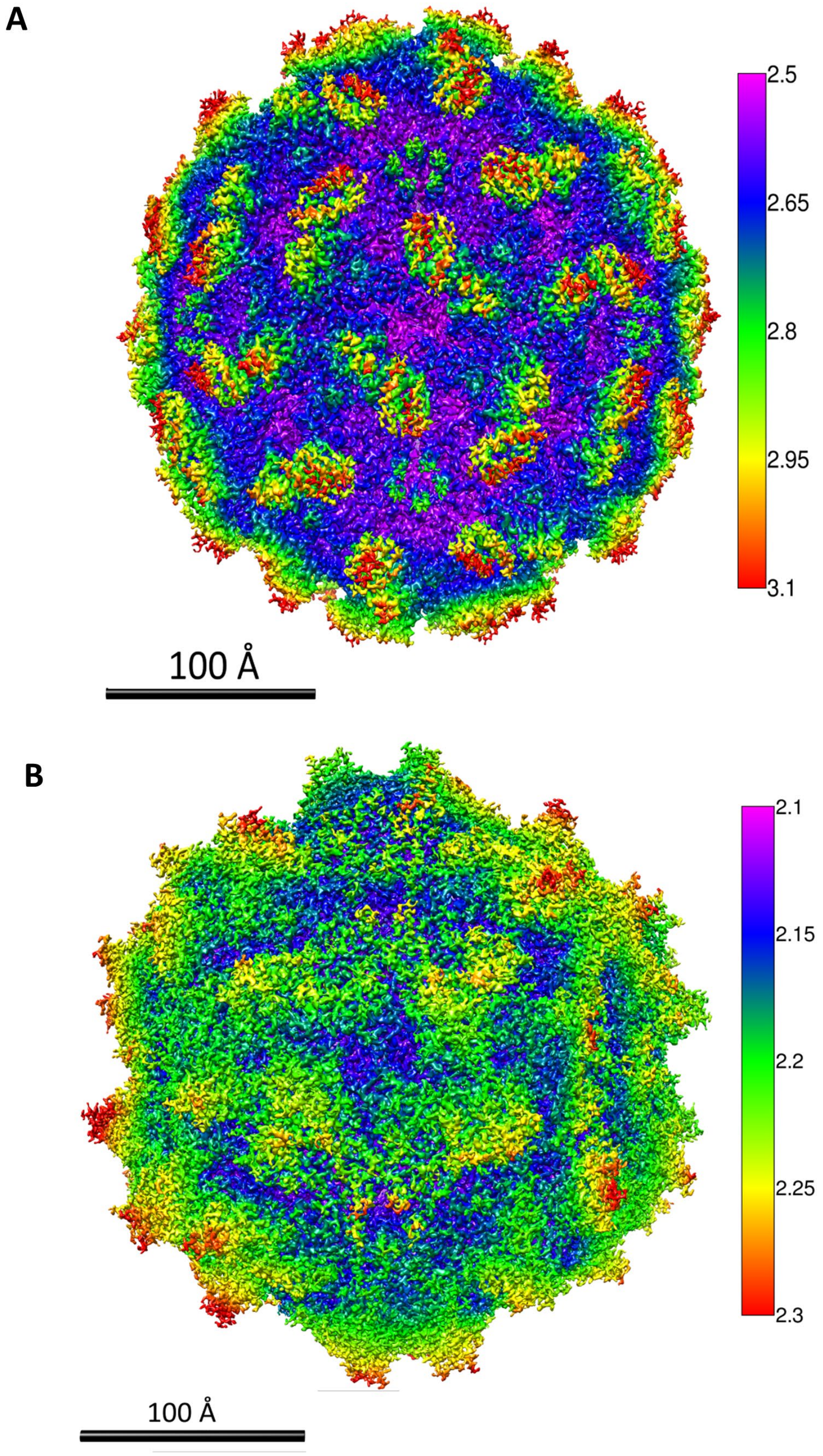

Figure S2. Coulombic potential maps colored by local resolution. Maps for both (**A**) AAV5 in complex with PKD1-2 and (**B)** uncomplexed AAV5 contoured at 1.6 sigma. The color keys are by resolution in Å

Supplementary Figure S3

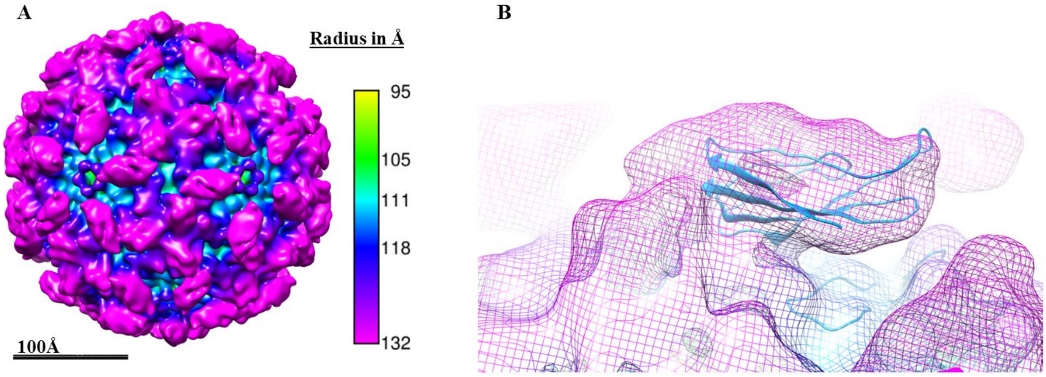

Figure S3. The 10 Å lowpass filtered cryo-EM coulombic potential map for AAV5 in complex with PKD1-2. (**A**) The view is along a 2-fold, with 2-fold axes also on horizontal and vertical axes. Three-fold axes can be seen above and below center, while 5-fold axes are left and right of center. The map is colored by distance from the center of the map. (**B**) A zoomed in image of A with a PKD1 domain inserted into the density. The lowpass filtered map shows the region of PKD1 close to the 5-fold axis of symmetry that cannot be seen at high resolution.

Supplementary Figure S4

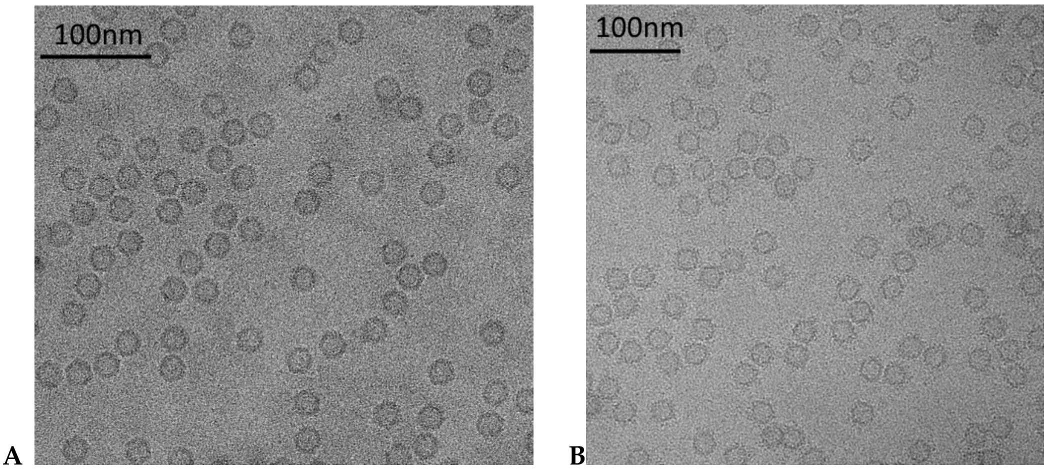

Figure S4. Typical Micrographs from the datasets. (**A**) Native AAV5 lowpass filtered at 20 Å shows VLPs in vitreous ice. (**B**) same as A but with AAV5-PKD1-2 bound

Supplementary Figure S5

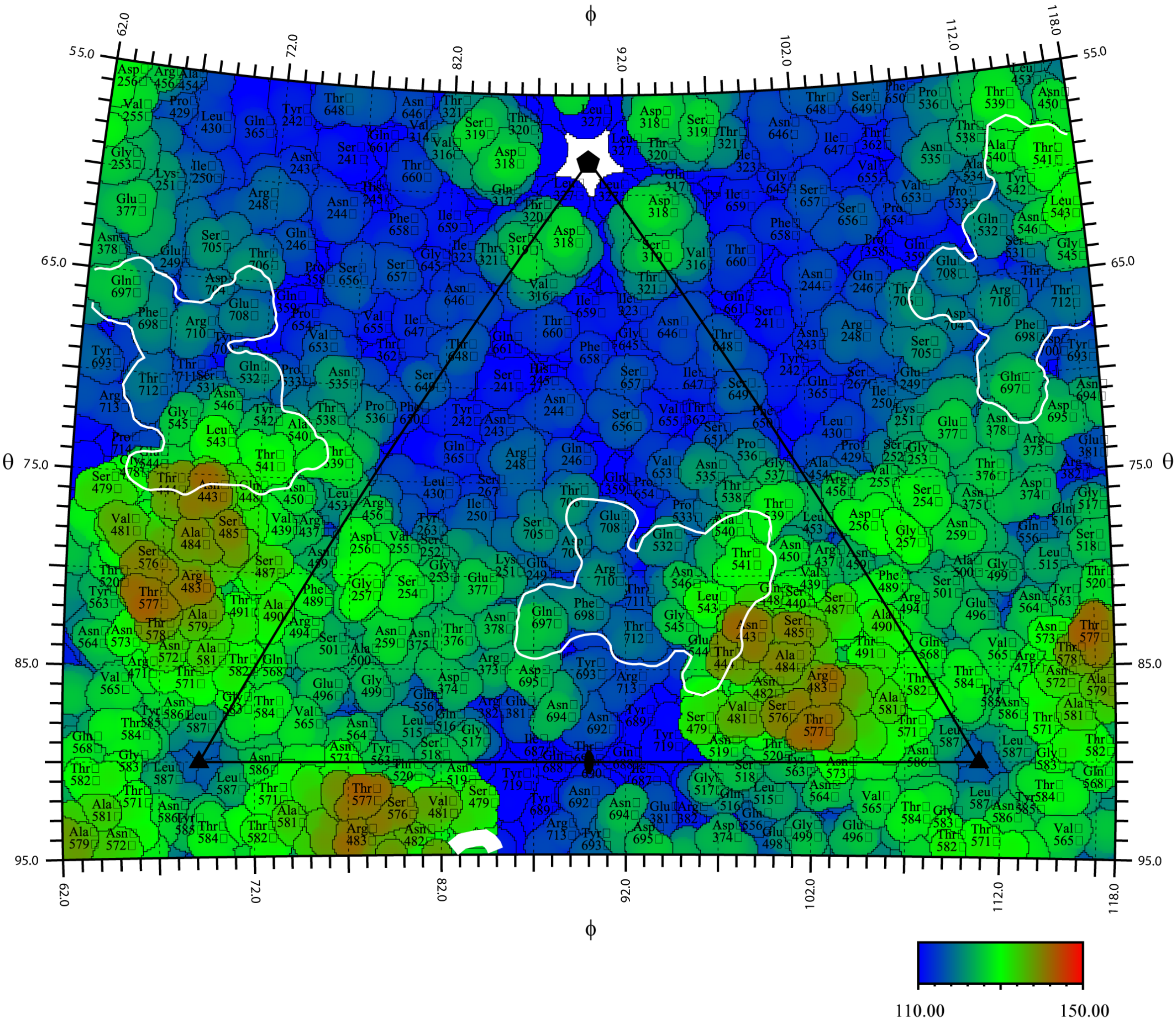

Figure S5 A full sized roadmap of PKD1 footprints on AAV5. Footprints of PKD1 are outlined in white and amino acids are labeled. Amino acids are colored by their distance from the center of the particle. Color scale is by distance in Å.

References

1. Zivanov, J.; Nakane, T.; Forsberg, B.O.; Kimanius, D.; Hagen, W.J.H.; Lindahl, E.; Scheres, S.H.W. New tools for automated high-resolution cryo-EM structure determination in RELION-3. *Elife* **2018**, *7*, 1–22, doi:10.7554/eLife.42166.

2. Chen, S.; McMullan, G.; Faruqi, A.R.; Murshudov, G.N.; Short, J.M.; Scheres, S.H.W.; Henderson, R. High-resolution noise substitution to measure overfitting and validate resolution in 3D structure determination by single particle electron cryomicroscopy. *Ultramicroscopy* **2013**, *135*, 24–35, doi:10.1016/j.ultramic.2013.06.004.

© 2020 by the authors. Submitted for possible open access publication under the terms and conditions of the Creative Commons Attribution (CC BY) license

(http://creativecommons.org/licenses/by/4.0/).
